# Correlation Between Liver Stiffness and Diastolic Function, Left Ventricular Hypertrophy, and Right Cardiac Function in Patients With Ejection Fraction Preserved Heart Failure

**DOI:** 10.3389/fcvm.2021.748173

**Published:** 2021-11-25

**Authors:** Junyi Zhang, Mingzhu Xu, Tan Chen, Yafeng Zhou

**Affiliations:** ^1^Department of Cardiology, Dushu Lake Hospital Affiliated to Soochow University, Suzhou, China; ^2^Department of Anesthesia, Dushu Lake Hospital Affiliated to Soochow University, Suzhou, China

**Keywords:** diastolic function, right cardiac function, liver stiffness, left ventricular hypertrophy, HFpEF

## Abstract

**Objective:** Ejection fraction preserved heart failure (HFpEF) is a common clinical syndrome with a high morbidity, accounting for ~50% of all heart failure patients, and a mortality comparable to that of ejection fraction reduced heart failure (HFrEF). The relationship between liver stiffness (LS) and HFpEF remains unclear. The purpose of this study was to explore the correlation between LS and the severity of HFpEF.

**Methods:** We performed a prospective observational study. After accepting liver transient elastography on admission, consecutive 150 hospitalized HFpEF patients were divided into three groups based on their liver elasticity value: first-third quartiles. Left ventricular diastolic function, left ventricular hypertrophy degree, right cardiac function and short-term prognosis (≤1 year) were compared among the three groups, and the correlation between liver elasticity and each indicator was analyzed.

**Results:** The elasticity of the liver was abnormally high in more than two-thirds of cases. The proportion of NYHA class III-IV in the third quartile group was significantly higher than that in the first quartile group (96 vs. 70%, *P* = 0.013). Significant differences were discovered in the level of lgNT-proBNP between the three groups (2.63 ± 0.65 vs. 2.84 ± 0.44 vs. 3.05 ± 0.71, *P* = 0.027). In terms of diastolic function and left ventricular hypertrophy, the ventricular septal e′ (5.01 ± 2.69 vs. 6.48 ± 2.29, *P* = 0.025), lateral wall e′ (6.63 ± 3.50 vs. 8.62 ± 2.73, *P* = 0.013), mean E/e′ (20.06 ± 7.53 vs. 13.20 ± 6.05, *P* = 0.001), left atrial volume index (43.53 ± 10.94 vs. 35.78 ± 13.86, *P* = 0.008), tricuspid regurgitation (TR) peak flow rate (3.16 ± 0.44 vs. 2.75 ± 0.50, *P* < 0.001), left ventricular mass index (LVMI) in male (163.2 ± 47.6 vs. 131.3 ± 38.0, *P* = 0.015) and in female (147.4 ± 48.6 vs. 110.6 ± 24.3, *P* = 0.036) was significantly different between the third quartile and the first quartile. The proportion of patients with diastolic dysfunction in the third quartile was significantly higher than that in the first quartile (70 vs. 36%, *P* = 0.017). In terms of right cardiac function, right ventricular fractional area change (RVFAC) (30.3 ± 5.4 vs. 36.5 ± 6.8, *P* < 0.001), tricuspid annular plane systolic excursion (TAPSE) (7.7 ± 5.2 vs. 14.8 ± 5.9, *P* = 0.010), pulmonary systolic pressure (38.0 ± 10.5 vs. 32.4 ± 10.3, *P* = 0.005), TR peak flow rate (3.16 ± 0.44 vs. 2.75 ± 0.50, *P* < 0.001), and inferior vena cava diameter (2.53 ± 0.51 vs. 1.98 ± 0.41, *P* < 0.001) were significantly different between the third quartile and the first quartile. More than half of HFpEF patients were combined with right ventricular dysfunction (RVD). Compared to HFpEF without RVD, HFpEF with RVD had higher male sex (53.6 vs. 30.3%, *P* < 0.001), higher NYHA class (3.2 ± 0.6 vs. 2.8 ± 0.6, *P* = 0.010), higher proportion of atrial fibrillation (45.2 vs. 18.2%, *P* < 0.001), and higher liver elasticity value (7.95 ± 0.60 vs. 7.31 ± 0.84, *P* = 0.003). In terms of short-term prognosis, the incidence of adverse cardiovascular events was significantly higher in the third quartile than in the first quartile (*P* = 0.003) and the second quartile (*P* = 0.008). Multivariate Cox proportional hazard analysis showed that adverse cardiovascular events were independently associated with NYHA class, atrial fibrillation, lgNT-proBNP and liver elasticity value (HR = 1.208, 95% CI 1.115–1.352, *P* = 0.002).

**Conclusion:** Increase of liver stiffness is common in HFpEF patients. Increased LS in HFpEF patients was significantly associated with worsen left diastolic function, left ventricular hypertrophy, and the right cardiac function. LS in HFpEF patients may be more than the result of right ventricular dysfunction. Male, atrial fibrillation, poorer NYHA class and increased liver elasticity value were significantly associated with HFpEF combined with RVD. Atrial fibrillation, poorer NYHA class, higher NT-proBNP, and increased liver elasticity value were independent predictors of poor short-term prognosis of HFpEF patients.

## Introduction

Heart failure preserved ejection fraction (HFpEF) is a specific type of heart failure with a normal left ventricular ejection fraction (LVEF), which is characterized by abnormal diastolic function, decreased compliance, and increased stiffness (1). HFpEF is a common and increasingly serious public health problem. It is estimated that HFpEF accounts for 40–50% of heart failure (HF) population, and its prevalence is increasing at an alarming rate of 1% per year compared to heart failure with reduced ejection fraction (HFrEF) (2). However, mortality of HFpEF is similar to HFrEF, and the risk of sudden death is even greater (3). Over the past decades, few studies have shown effective treatments for HFpEF, making HFpEF a growing public health problem.

Heart failure, as a systemic disease, is often accompanied by impaired functional reserve of multiple organs, such as lung, blood vessels, skeletal muscle, kidney and liver (4, 5). Many studies have shown that congestive liver disease caused by heart failure can lead to liver dysfunction and increase liver stiffness (LS). Abnormal liver in turn aggravates the clinical prognosis of patients with heart failure, namely the so-called “heart-liver syndrome” (6, 7). As a special type of heart failure, HFpEF should be regarded as a systematic disease due to its complexity and diversity of pathophysiological mechanisms (8), making its relationship with possible secondary liver dysfunction and liver fibrosis more complex. However, up to now, few studies have reported the relationship between HFpEF and LS.

Inflammation plays an important role in the pathophysiological process of HFpEF (9). HFpEF is a state of relaxation and sclerosis of the myocardial and arterial systems (10), which is driven by a systemic proinflammatory state caused by a variety of non-cardiac comorbidities. Proinflammatory state causes vascular endothelial inflammation in coronary arteries, inhibits the NO-cGMP-protein kinase G pathway in adjacent cardiomyocytes (11), promotes cardiomyocyte hypertrophy, increases resting tension of cardiomyocytes, and increases myocardial collagen deposition by proliferating fibroblasts and myofibroblasts. Secondary cardiomyocyte stiffness and interstitial fibrosis lead to centripetal left ventricular remodeling and diastolic dysfunction of HFpEF. Similarly, systematic inflammation activates liver macrophages, causes autophagy apoptosis of liver cells, increases liver injury and secondary liver fibrosis (12). In fact, many subgroups of HFpEF such as metabolic syndrome, insulin resistance, obesity are also at high risk of increased LS (13, 14). Therefore, HFpEF may be more likely to increase LS, and the degree of LS may have some inherent correlation with heart failure severity.

In the preliminary experiment, 30 HFrEF and 30 HFpEF patients were matched by sex and age 1:1 for liver elastography measurement (data seen in Supplementary Materials). The results suggested that there was obvious increased LS in both groups. However, no significant difference was found between the two groups, and the liver elasticity value was even higher in HFpEF than that of HFrEF (Supplementary Table 1). This suggests that elevated LS in HFpEF may be independent of left ventricular ejection fraction (LVEF). So we asked a question: since HFpEF is characterized by diastolic dysfunction and decreased cardiac compliance, is the degree of LS inherently associated with diastolic function and ventricular remodeling?

Liver biopsy is the gold standard for diagnosing liver fibrosis, but it is an invasive and expensive test that carries a risk of bleeding, pneumothorax, hemothorax or damage to adjacent organs. In recent years, elastography, as an emerging, non-invasive and high-accuracy method to measure the stiffness of parenchymal organs, has been increasingly used in the assessment of LS (15).

The purpose of this study was to evaluate the relationship between LS and left ventricular diastolic function, left ventricular hypertrophy, right cardiac function and short-term prognosis in patients with HFpEF.

## Materials and Methods

### Subjects and Study Protocol

This was a prospective observational study that enrolled patients hospitalized for treatment of HFpEF and discharged from the First Affiliated Hospital of Soochow University between 2019 and 2020. HFpEF was defined based on the following criterias: (1) have typical signs or symptoms of heart failure; (2) LVEF ≥ 50%; (3) B-type natriuretic peptide (BNP) > 35 ng/L and/or N-terminal B-type natriuretic peptide precursor (NT-proBNP) > 125 ng/L. In addition, they met at least one of the following criterias: (1) evidence of left ventricular hypertrophy and/or left atrial enlargement, and (2) evidence of abnormal diastolic function. Exclusion criteria were as follows: (1) patients with acute heart failure, acute myocardial infarction, acute myocarditis, pericardial disease, and congenital heart disease; (2) patients with clear liver diseases, including various hepatitis and various cirrhosis with definite etiology (e.g., viral hepatitis cirrhosis, schistosomiasis cirrhosis, alcoholic cirrhosis, cholestasis cirrhosis, autoimmune cirrhosis and toxic/drug cirrhosis); (3) patients with advanced tumor; (4) patients requiring dialysis.

Blood samples and echocardiography were obtained at admission (echocardiography machine: EPIQ 7C, Philips, Netherland). In addition, all patients accepted liver elastography at admission. The patients were divided into three groups according to the measured liver elasticity value (Elastic PQ) by the trinity method: first quartile (Elastic PQ < 7.15 kPa, *n* = 50), second quartile (7.15 kPa ≤ Elastic PQ < 8.30 kPa, *n* = 50) and third quartile (8.30 kPa ≤ Elastic PQ, *n* = 50).

### Measurement of LVEF

LVEF is calculated by Teichholz method: left ventricular end-diastolic dimension (LVDd) and left ventricular end-systolic dimension (LVDs) were measured during the cardiac cycle. Then, use the corrected cubic formula V = (2.4 + D)^π^D^3^ (this formula imagines the left ventricular volume as an approximate ellipsoid) to calculate the left ventricular end-diastolic volume (V_d_) and end-systolic volume (V_s_), respectively. LVEF was then calculated by the formula LVEF = (V_d_ – V_s_)/${\text{V}}_{\text{d}}^{*}$100%. Of course, in actual operation, the operator only needs to measure LVDd and LVDs, and the rest is calculated automatically by the machine.

### Measurement of Liver Elastography

**Figure 1:**
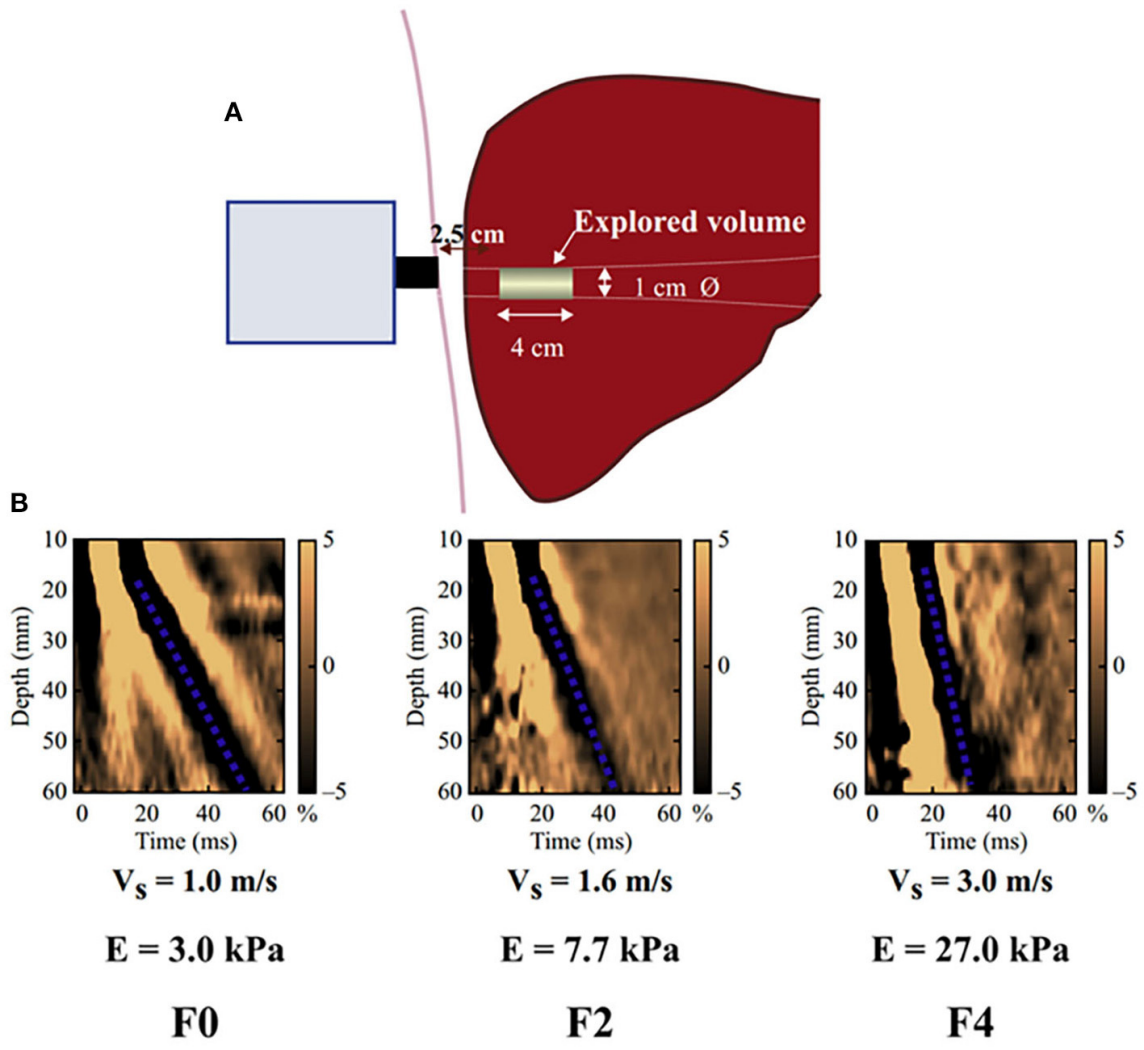
Schematic diagram of the basic principles of hepatic transient elastography. **(A)** Schematic diagram of basic principles of transient elastic imaging of liver. **(B)** Judging the severity of liver fibrosis according to shear wave propagation velocity. The elastic modulus E is expressed as E = 3ρV2, where V is the shear velocity and ρ is the mass density (constant and close to 1 kg/m^3^ for the tissue): the harder the tissue, the faster the shear wave propagates. In the absence of fibrosis (F0), the speed was 1.0 m/s and the elasticity was 3 kPa, while in the case of cirrhosis (F4), the speed was 3.0 m/s and the elasticity was 27 kPa.

In this study, the liver elasticity value measured by transient elastography (TE) was used as the indicator to evaluate LS. LS measurements were performed by a single experienced examiner (Xu. MZ) at admission, who was blinded to all clinical data, using an ultrasonic elastography machine (SonixTouch, Ultrasonix, Canada). In particular, TE is performed on a patient lying supine, with the right arm elevated to facilitate access to the right liver. The tip of the probe is in contact with the intercostal skin through a coupling gel in the 9th to 11th intercostal space at the level where TE would be performed. The operator, assisted by a time-motion image, locates a liver portion at least 6 cm deep and free of large vascular structures. The operator then presses the probe button to start the measurements (“shots”). TE measures the liver stiffness in a volume that approximates a cylinder 1 cm wide and 4 cm long, between 25 and 65 mm below the skin surface (Figure 1). The software determines whether each measurement is successful or not. When a shot is unsuccessful, the instrument does not return a value. The entire procedure is considered to have failed when no values are obtained after 10 shots. Successful measurements are validated using the following criteria: (1) number of valid shots ≥ 10; (2) ratio of valid shots to the total number of shots ≥60%; and (3) interquartile range (IQR, reflecting the variability of measurements) <30% of the median liver stiffness measurement (LSM) value (IQR/LSM ≤ 30%) (15, 16).

TE is a patient-friendly procedure: it only requires a short time (5 min) and can be performed bedside. The results are expressed in kilopascals (kPa), ranging from 2.5 to 75 kPa, and are available immediately. After recording 10 measurements, the average value is taken as the final measurement result.

### Indicators of Left Ventricular Diastolic Function

According to “Recommendations for the Evaluation of Left Ventricular Diastolic Function by Echocardiography: An Update from the American Society of Echocardiography and the European Association of Cardiovascular Imaging” (17), the guideline team recommended four indicators and their critical values for the identification of left ventricular diastolic function as follows: The e′ velocity of mitral annulus (ventricular septum e′ < 7 cm/s, lateral wall e′ < 10 cm/s), mean E/e′ > 14, left atrial volume index > 34 mL/m^2^, tricuspid regurgitation (TR) peak flow rate > 2.8 m/s. Therefore, ventricular septum e′, lateral wall e′, mean E/e′, left atrial volume index, and TR peak flow rate were used as indicators to evaluate left ventricular diastolic function in our study. All the above data were obtained by echocardiography. Then, according to the algorithm of left ventricular diastolic dysfunction in patients with normal LVEF developed by the guidelines writing group, the patients were judged for diastolic dysfunction (17).

### Indicators of Left Ventricular Hypertrophy

According to “Chinese Guidelines for the Diagnosis and Treatment of Heart Failure 2018” (18), left ventricular mass index (LVMI) is the main indicator of left ventricular hypertrophy, and left ventricular mass index ≥ 115 g/m^2^ (male) or 95 g/m^2^ (female) is considered to have significant left ventricular hypertrophy. LVMI can be calculated from cardiac data and body surface area: LVM = (IVST + LVDd + PWT) 3-LVDd3; LVMI = LVM/BSA (LVM = left ventricular mass; BSA = body surface area; IVST = interventricular septal thickness; LVDD = left ventricular end-diastolic diameter; PWD = diastolic left ventricular posterior wall thickness; BSA = body surface area) (19).

### Indicators of Right Cardiac Function

Up to now there is no uniform indicators for the evaluation of right cardiac function. In this study, we used the following indicators recommended by the Heart Failure Association of the European Society of Cardiology (20) to evaluate the right cardiac function of HFpEF: right ventricular fractional area change (RVFAC), tricuspid annular plane systolic excusion (TAPSE), pulmonary systolic pressure, TR peak flow rate and inferior vena cava diameter. TAPSE and RVFAC were used to evaluate right ventricular systolic function, while the remaining indicators were used to evaluate right ventricular pressure/volume load. All the above data were obtained by echocardiography.

### Indicators of Short-Term Prognosis

Short-term prognosis is reflected by major adverse cardiovascular event (MACE) in 1 year after discharge, including cardiovascular death, malignant arrhythmia, myocardial infarction, stroke and re-hospitalization due to heart failure.

### Statistical Analysis

The measurement data were tested for normality and homogeneity of variance. measurement data obeying normal distribution and homogeneous population variance are presented with mean ± standard deviation, otherwise, logarithmic conversion was carried out and expressed in the form of lg (such as NT-proBNP). Since this study was a comparison among three groups, one-way analysis of variance and Kruscal-Wallis H test were used to compare the measurement data between groups according to whether they were in line with normal distribution and homogeneity of variance. For counting data, it is expressed as frequency and percentage (*n*, %). Chi-square test was used to compare the counting data. If the frequency is <5, Fisher's exact-test is used. Spearman correlation analysis was used to analyze the correlation between liver elasticity value and diastolic function indicators, left ventricular remolding indicators and right cardiac function indicators. Statistics were conducted using SPSS 24.0. *P* < 0.05 was considered statistically significant.

## Results

### Comparison of Basic Characteristics

**Figure 2:**
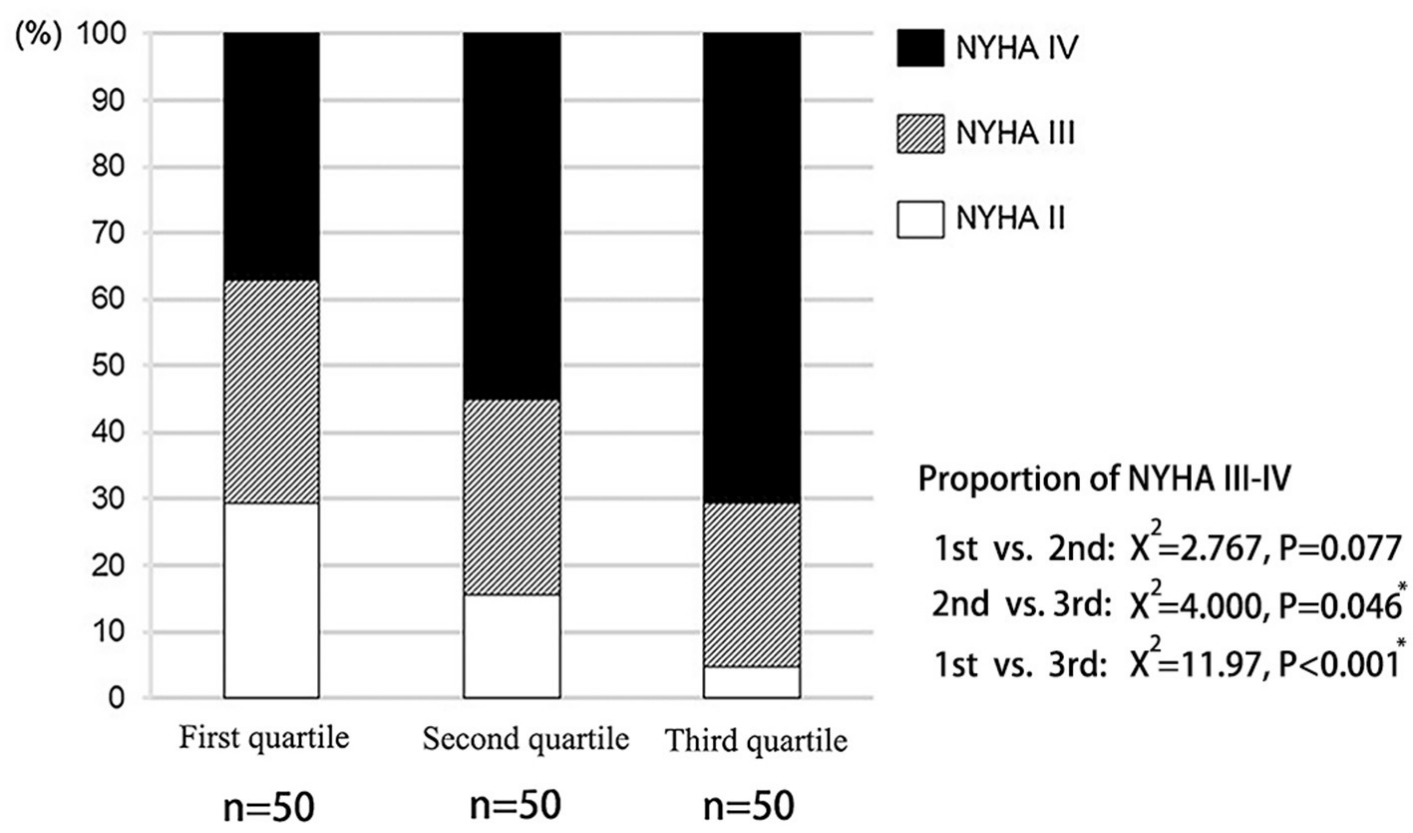
Distribution of cardiac function in patients with different liver hardness groups.

The clinical features of the present study's subjects are summarized in Supplementary Table 2. The third quartile group had a higher prevalence of diabetes and atrial fibrillation. The proportion of NYHA III-IV in the third quartile was also significantly higher than that of other groups (Figure 2). However, there was no significant difference in LVEF among the three groups. Although LVDd increased from the first to the third quartile groups, there was no statistical difference. There was no statistical difference in the prevalence of patients with hypertension, hyperlipidemia, coronary heart disease, chronic kidney disease, chronic obstructive pulmonary disease and anemia among the three groups.

In terms of laboratory data, as the overall value of NT-proBNP does not meet the normal distribution (*P* = 0.034), we take the logarithm of the NT-proBNP and then compare it between groups. As a result, the third quartile group had the highest lgNT-proBNP, as well as levels of abumin/globulin ratio (A/G), total bilirubin, direct bilirubin and indirect bilirubin. In contrast, ALT, AST, alkaline phosphatase (ALP), γ-glutamyl transferase (GGT), lipid levels and the electrolyte did not differ significantly among the three groups.

Spearman correlation analysis was performed between liver elasticity value and laboratory data. The results showed that the ratio of liver elasticity value to blood A/G (*R* = −0.235, *P* = 0.020), total bilirubin (*R* = 0.325, *P* = 0.001), direct bilirubin (*R* = 0.497, *P* < 0.001), indirect bilirubin (*R* = 0.308, *P* = 0.002), ALP (*R* = −0.215, *P* = 0.013), GGT (*R* = 0.220, *P* = 0.029), and lgNT-proBNP (*R* = 0.354, *P* < 0.001) were significantly correlated. Scatter distribution of liver elasticity values and various parameters is shown in Supplementary Figure 1.

### Comparison of Left Ventricular Diastolic Function and Left Ventricular Hypertrophy Indicators

Ventricular septum e′, lateral wall e′, mean E/e′, left atrial volume index (LAVI), TR peak flow rate and left ventricular mass index (LVMI) were compared among the three groups. As a result, ventricular septum e′ and lateral wall e′ were lower in the third quartile than in the first quartile (*P* = 0.025; *P* = 0.013). The higher the liver elasticity value, the higher the mean E/e′, and there were statistical differences between groups (first quartile vs. second quartile: *P* = 0.032; second quartile vs. third quartile: *P* = 0.019; first quartile vs. third quartile: *P* = 0.001). The LAVI of the first, second and third quartile groups showed an increasing trend. There was a statistical difference between the first quartile group and the third quartile group (*P* = 0.008), as well as between the second quartile group and the third quartile group (*P* = 0.044). The higher the liver elasticity value, the higher the TR peak flow rate, and there was significant difference among all groups (first quartile vs. second quartile: *P* = 0.048; second quartile vs. third quartile: *P* = 0.025; first quartile vs. third quartile: *P* < 0.001) (21). The LVMI of each patient was calculated, and the results showed that the LVMI in the third quartile was significantly higher than that in the first quartile, both in male and female (*P* = 0.015; *P* = 0.036). The comparison of the above data is shown in Table 1.

**Table 1:** Comparison of left ventricular diastolic function and left ventricular remodeling indexes in HFpEF patients with different liver hardness groups.

	**First quartile (*N* = 50)**	**Second quartile (*N* = 50)**	**Third quartile (*N* = 50)**	***P-*value**
Ventricular septal e′ (cm/s)	6.48 ± 2.29	5.73 ± 1.40	5.01 ± 2.69^*^	0.030
Lateral wall e′ (cm/s)	8.62 ± 2.73	7.52 ± 1.76	6.63 ± 3.50^*^	0.037
Average E/e′	13.20 ± 6.05^#^	16.54 ± 5.27^*^	20.06 ± 7.53^*^^#^	0.001
LAVI (mL/m^2^)	35.78 ± 13.86	38.57 ± 10.30	43.53 ± 10.94^*^^#^	0.031
TR peak flow rate (m/s)	2.75 ± 0.50^#^	2.95 ± 0.53^*^	3.16 ± 0.44^*^^#^	0.015
**LVMI (g/m** ^**2**^ **)**				
Male	131.3 ± 38.0	144.7 ± 24.5	163.2 ± 47.6^*^	0.016
Female	110.6 ± 24.3	125.9 ± 21.2	147.4 ± 48.6^*^	0.029

*LAVI, left atrial volume index; TR, Tricuspid regurgitation; LVMI, left ventricular mass index*.

* *P < 0.05 vs. the first quartile*.

# *P < 0.05 vs. the second quartile*.

Spearman correlation analysis was performed between liver elasticity values and left ventricular diastolic function indicators and left ventricular hypertrophy indicators. The results showed that liver elasticity value was negatively correlated with ventricular septum e′ and lateral wall e′, but positively correlated with mean E/e′, LAVI, TR peak flow rate and LVMI (Table 2). Scatter distribution of liver elasticity values and various parameters is shown in Supplementary Figure 2.

**Table 2:** Spearman correlation analysis of liver elasticity value with left ventricular diastolic function parameters and left ventricular remodeling indexes.

	**X ± s**	***R-*value**	***P-*value**
Ventricular septal e′ (cm/s)	6.12 ± 2.28	−0.253	0.012
Lateral wall e′ (cm/s)	7.79 ± 3.16	−0.260	0.010
Mean E/e′	16.11 ± 6.37	0.420	<0.001
LAVI (mL/m^2^)	37.92 ± 9.59	0.412	<0.001
TR peak flow rate (m/s)	2.91 ± 0.42	0.371	<0.001
**LVMI (g/m** ^**2**^ **)**			
Male	146.19 ± 34.51	0.511	<0.001
Female	124.20 ± 24.75	0.417	0.002

*LAVI, left atrial volume index; TR, Tricuspid regurgitation; LVMI, left ventricular mass index*.

**Figure 3:**
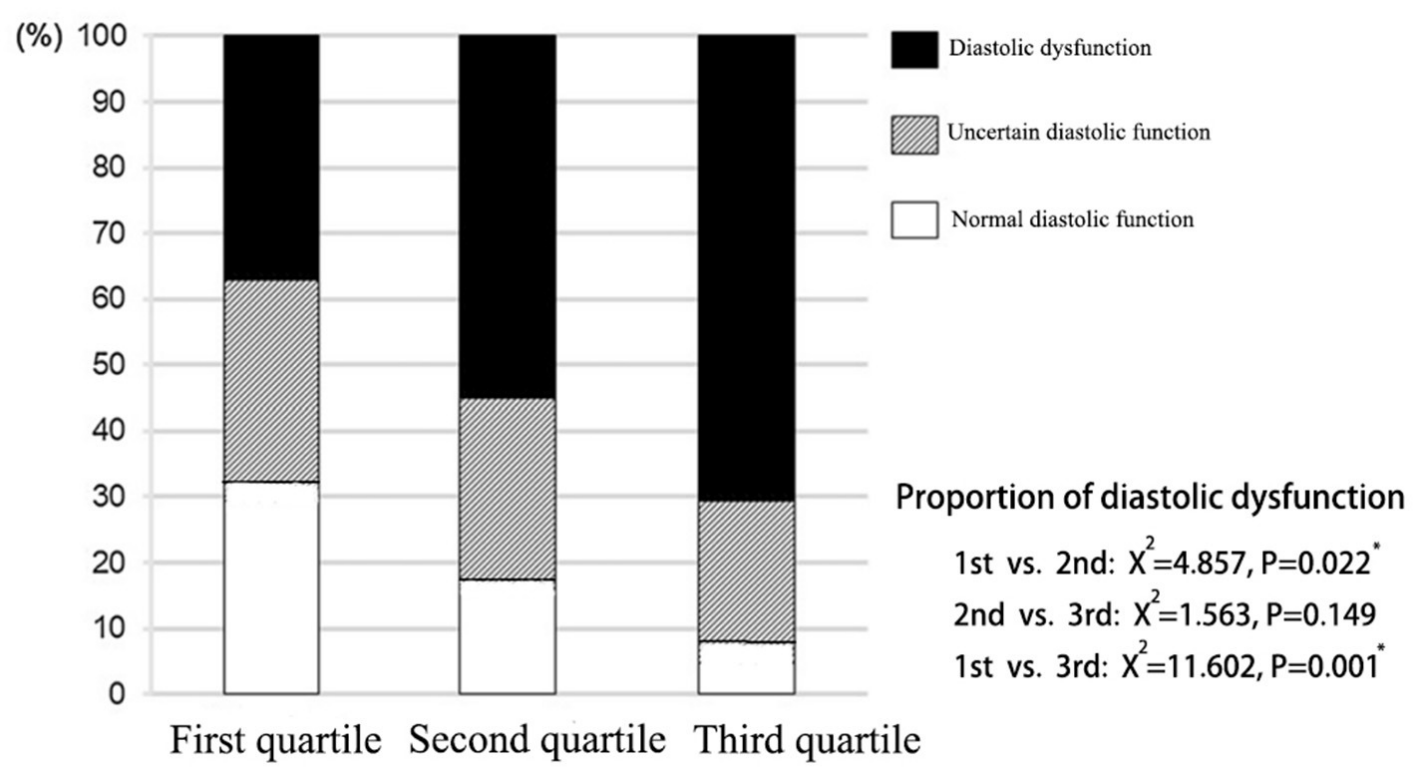
Distribution of left ventricular diastolic function in patients with different liver hardness groups.

According to the assessment criteria of left ventricular diastolic dysfunction as shown in section Method, the left ventricular diastolic function of each patient was assessed, and the number of patients with left ventricular diastolic dysfunction in each group was compared (Table 3). As a result, the proportion of patients with normal diastolic function in the three groups showed a decreasing trend, and the proportion of patients with normal diastolic function in the third quartile was significantly lower than that in the first quartile (*P* = 0.008). On the contrary, proportion of patients with diastolic dysfunction in the third quartile was significantly higher than that in the first quartile (*P* = 0.017; Figure 3).

**Table 3:** Comparison of the left ventricular diastolic function among groups.

	**First quartile** ***N* = 50**	**Second quartile** ***N* = 50**	**Third quartile** ***N* = 50**	***P-*value**
Normal diastolic function (*n*, %)	16 (32)	9(18)	4(8)^*^	**0.015**
Uncertain diastolic function (*n*, %)	16 (32)	12(24)	11(22)	0.104
Diastolic dysfunction (*n*, %)	18 (36)	29(58)	35(70)^*^	**0.026**

* *P < 0.05 vs. the first quartile*.

*Bold values indicates that the overall p value is statistically significant (<0.05)*.

### Comparison of Right Cardiac Function Indicators

With regard to the right heart echocardiographic parameters, although the right atrial diameter and right ventricular diameter did not differ among the three groups, indicators of right ventricular systolic function, such as RVFAC and TAPSE were smaller in the third quartile than in the first quartile (*P* < 0.001; *P* = 0.010). In addition, indicators of right heart volume/pressure, including pulmonary systolic pressure, TR peak flow rate and inferior vena cava (IVC) diameter all showed inter-group differences. Specifically, the pulmonary systolic pressure of the third quartile was significantly higher than that of the first and second quartile (*P* < 0.001; *P* = 0.016). TR peak flow rate differed significantly among all groups. The higher the liver elasticity value was, the greater the TR peak flow rate was (first quartile vs. second quartile: *P* = 0.031; second quartile vs. third quartile: *P* = 0.020; first quartile vs. third quartile: *P* < 0.001). The higher the liver elasticity value was, the larger the IVC diameter was, and the IVC diameter in the third quartile was significantly greater than that in the first quartile (*P* < 0.001; Table 4).

**Table 4:** Comparison of right heart function in HFpEF patients of different liver hardness groups.

	**First quartile** **(*N* = 50)**	**Second quartile** **(*N* = 50)**	**Third quartrile** **(*N* = 50)**	***P-*value**
Right atrial diameter (mm)	37.1 ± 5.3	39.3 ± 8.3	40.1 ± 8.4	0.282
Right ventricular diameter (mm)	34.4 ± 0.9	34.9 ± 0.9	35.7 ± 0.9	0.577
RVFAC (%)	36.5 ± 6.8	33.4 ± 5.7	30.3 ± 5.4^*^	**0.007**
TAPSE (mm)	14.8 ± 5.9	11.5 ± 5.5	7.7 ± 5.2^*^	**0.019**
Pulmonary systolic pressure (mmHg)	39.3 ± 10.1	44.8 ± 7.2	50.9 ± 10.4^*^^#^	**0.008**
TR peak flow rate (m/s)	2.75 ± 0.50^#^	2.95 ± 0.53^*^	3.16 ± 0.44^*^^#^	**0.015**
IVC diameter (cm)	1.98 ± 0.41	2.20 ± 0.45	2.53 ± 0.51^*^	**0.016**

*RVFAC, right ventricular fractional area change; TAPSE, tricuspid annular plane systolic excusion; TR, tricuspid regurgitation; IVC, inferior vena cava*.

* *P < 0.05 vs. the first quartile*.

# *P < 0.05 vs. the second quartile. Bold values indicates that the overall p value is statistically significant (<0.05)*.

Spearman correlation analysis was performed between liver elasticity values and right cardiac function indicators in all patients. As a result, liver elasticity value was significantly negatively correlated with RVFAC and TAPSE, and positively correlated with right atrial diameter, pulmonary systolic pressure, and TR peak flow rate, but not significantly correlated with right ventricular diameter and IVC diameter (Table 5). Scatter distribution of liver elasticity values and the above parameters is shown in Supplementary Figure 3.

**Table 5:** Spearman correlation analysis of liver elasticity value with right heart function parameters.

	**X ± s**	***R*-value**	***P-*value**
Right atrial diameter (mm)	39.50 ± 7.68	0.205	**0.034**
Right ventricular diameter (mm)	34.92 ± 5.11	0.118	0.246
RVFAC (%)	36.22 ± 6.09	−0.398	**<0.001**
TAPSE (mm)	12.06 ± 5.42	−0.306	**0.002**
Pulmonary systolic pressure (mmHg)	45.33 ± 9.50	0.434	**<0.001**
TR peak flow rate (m/s)	2.95 ± 0.51	0.566	**<0.001**
IVC diameter (cm)	2.31 ± 0.48	0.296	0.078

*RVFAC, right ventricular fractional area change; TAPSE, tricuspid annular plane systolic excusion; TR, tricuspid regurgitation; IVC, inferior vena cava. Bold values indicates that the overall p value is statistically significant (<0.05)*.

According to the cardiac ultrasound criteria for right heart dysfunction (RVD) of HFpEF established by the Heart Failure Association of the European Society of Cardiology, RVD exists when any of the following criteria is satisfied: RVFAC < 35%, TAPSE < 17 mm, TR peak flow rate > 2.8 m/s, IVC diameter > 21 mm, IVC collapse index < 50% at the end of inspiration. According to this criterion, 84 of the 150 patients in this study were combined with RVD, while the others were not. The liver elasticity value and basic data of the two groups were compared (Table 6). As a result, the liver elasticity value of the RVD group was significantly higher than that of the no-RVD group (7.95 ± 0.60 vs. 7.31 ± 0.84, *P* = 0.003). In addition, proportion of male, NYHA class, and proportion of atrial fibrillation in the RVD group were significantly higher than those in the no-RVD group (*P* < 0.001, *P* = 0.01, *P* < 0.001).

**Table 6:** Comparison of clinical data in HFpEF patients with or without RVD.

	**HFpEF without RVD** **(*N* = 66)**	**HFpEF with RV** **(*N* = 84)**	***P-*value**
Age (years)	67.3 ± 13.4	68.9 ± 13.6	0.232
Male (*n*, %)	20 (30.3)	45 (53.6)	**<0.001**
LVEF (%)	62.4 ± 12.5	60.3 ± 12.1	0.253
NYHA class	2.8 ± 0.6	3.2 ± 0.6	**0.01**
Hypertension (*n*, %)	46 (69.7)	60 (71.4)	0.424
Diabetes (*n*, %)	21 (31.8)	29 (34.5)	0.296
Hyperlipidemia (*n*, %)	13 (19.7)	20 (23.8)	0.403
CAD (*n*, %)	14 (21.2)	26 (31.0)	0.062
Atrial fibrillation (*n*, %)	12 (18.2)	38 (45.2)	**<0.001**
LEV (kPa)	7.31 ± 0.84	7.95 ± 0.60	**0.003**

*RVD, right ventricular dysfunction; LVEF, left ventricular ejection fraction; CAD, coronary artery disease; LEV, liver elasticity value. Bold values indicates that the overall p value is statistically significant (<0.05)*.

### Comparison of Short-Term Prognosis

All the patients were followed up, with a maximum of 386 days, a minimum of 52 days, and a median of 197 days. There was no statistical difference in the median follow-up time among the three groups. Due to the low incidence of cardiogenic death, malignant arrhythmia, acute myocardial infarction and stroke (*N* < 5 in group), the chi-square test was not performed for these outcomes. However, significant differences were detected in the incidence of hospitalization due to heart failure and total number of major cardiovascular events (MACE) among groups (Table 7). The incidence of hospitalization due to heart failure in the third quartile was significantly higher than that in the first quartile (*X*^2^ = 5.482, *P* = 0.019). Total number of MACE in the third quartile was significantly higher in the third quartile than that in the first (*X*^2^ = 9.653, *P* = 0.003) and second quartile (*X*^2^ = 7.104, *P* = 0.008).

**Table 7:** Comparison of short-term (<1 year) prognosis of HFpEF patients of different liver hardness groups.

**MACE**	**First quartile** **(*N* = 50)**	**Second quartile** **(*N* = 50)**	**Third quartile** **(*N* = 50)**	***X* ^**2**^**	***P-*value**
Median follow-up time (month)	7.2	7.5	7.4	-	0.935
Cardiovascular death (*n*, %)	1 (2)	1 (2)	3 (6)	-	-
Malignant arrhythmia (*n*, %)	0 (0)	1 (2)	1 (2)	-	-
AMI (*n*, %)	1 (2)	0 (0)	3 (6)	-	-
Stroke (*n*, %)	1 (2)	2 (4)	2 (4)	-	-
Hospitalization due to heart failure (*n*, %)	7 (14)	11 (22)	17 (34)^*^	5.665	**0.039**
**Total**	11 (22)	13 (26)	26 (52)^*^^#^	11.943	**0.003**

*AMI, acute myocardial infarction*.

* *P < 0.05 vs. the first quartile*.

# *P < 0.05 vs. the second quartile. Bold values indicates that the overall p value is statistically significant (<0.05)*.

**Figure 4:**
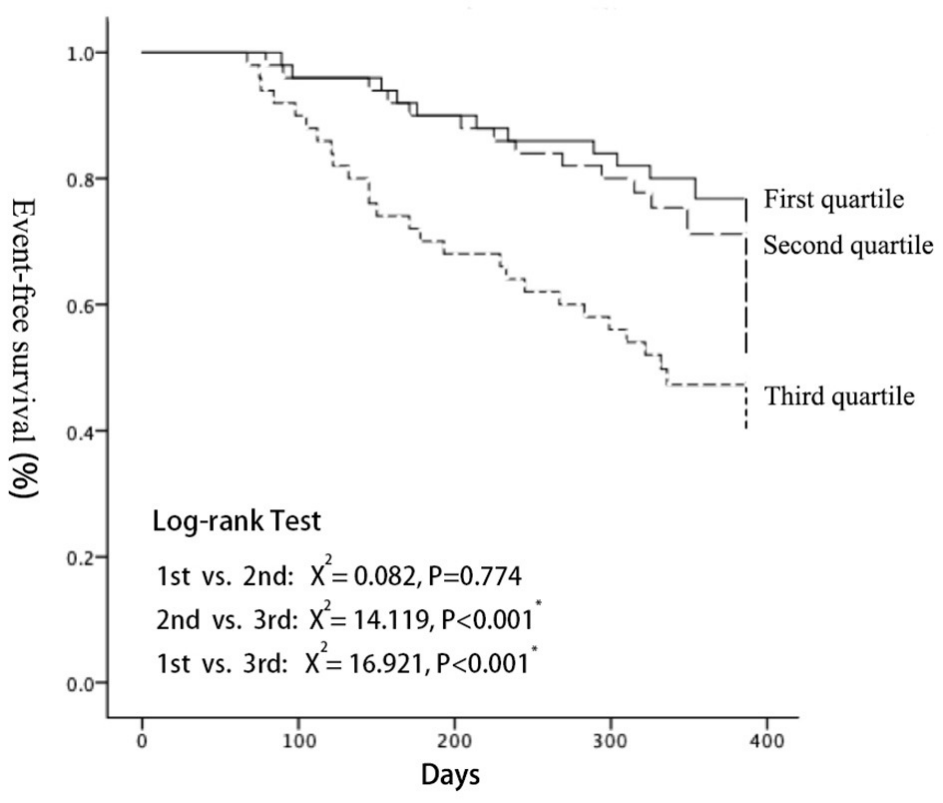
Kaplan-Meier analyses demonstrated that a significantly higher cardiac event rate was observed with increasing LEV (log-rank test: *P* < 0.001). LEV, liver elastography value.

MACE-free Kaplan Meier (K-M) survival curves were drawn for the three groups, and the difference of the survival curves among groups were compared using the Log-Rank Test (Figure 4). As a result, with the increase of follow-up days, the MACE-free survival rate among groups differed. The MACE-free survival rate in the third quartile was significantly lower than that in the first (*X*^2^ = 15.044, *P* < 0.001) and the second quartile (*X*^2^ = 14.119, *P* < 0.001). There was no statistical difference between the first quartile and the second quartile (detailed data seen in Supplementary Figure 4).

The Cox proportional hazard model was used to examine the prognostic value of the liver elasticity value in HFpEF patients. Patients were divided into event group and non-event group according to whether MACE occurred during follow-up. Univariate Cox regression analysis showed that age, BMI, NYHA class, diabetes mellitus, atrial fibrillation, smoking, eGFR, lg NT-proBNP, hemoglobin, albumin, LDL-C, and LEV were associated with MACEs (unadjusted hazard ratio 1.305, 95% CI 1.152–1.485, *P* < 0.0001). In the Multivariate analysis, increased LEV was independently associated with MACEs after adjusting for NYHA class, atrial fibrillation and lgNT-proBNP (adjusted hazard ratio 1.210, 95% CI 1.117–1.353, *P* = 0.00204, Supplementary Table 3).

## Discussion

The effect of heart failure on the liver has been widely discussed in recent years. The liver receives a dual blood supply from the portal vein and the hepatic artery. Blood from the portal vein and the hepatic artery mingle with the hepatic sinusoid, converged from the central vein into the hepatic vein, and then diverted into the inferior vena cava and finally into the right atrium. Stasis liver disease is very common in patients with heart failure. Decrease of cardiac output leads to increased left atrial pressure, obstruction of pulmonary venous blood entering the left atrium, increased pulmonary circulation pressure, obstruction of right ventricle pumping blood to the pulmonary artery, and further overload of right ventricular pressure capacity, eventually leading to increased central venous pressure (20). Liver congestion is not the only cause of liver injury caused by heart failure, but also the decrease of hepatic arterial blood flow. Insufficiency of hepatic artery blood supply can cause hypoxia, atrophy and necrosis of liver cells, and secondary destruction of liver tissue, which is called hypoxic liver disease (22). In the state of persistent heart failure, necrosis of the lobular central hepatocytes may extend to the surrounding area with deposition of connective tissue connecting the affected central vein, eventually leading to increased LS and even cirrhosis (22–24).

However, few researches have been done on the effects of HFpEF on liver. Cardiac systolic function of HFpEF is usually normal or only mildly impaired, but widespread diastolic dysfunction can also lead to left ventricular pressure volume overload. Theoretically, it can also lead to liver congestion, which in turn increases LS. For the first time, our study found that the degree of LS in patients with HFpEF was strongly correlated with its left ventricular diastolic function, left ventricular hypertrophy, right cardiac function and short-term prognosis. In other words, the degree of LS in patients with HFpEF could reflect the characteristics and severity of the disease.

In this study, liver elastography was used as a means to detect LS The degree of LS cannot be accurately judged by ordinary two-dimensional ultrasound, while the emergence of liver elastography has made up for this deficiency. Ultrasonic elastography is a method to measure the hardness of tissue. Different hardness of tissue has different elastic coefficients. Elastography has unique advantages in evaluating the complications of cirrhosis and portal hypertension. The subjects of this study were patients with heart failure, who were generally unable to tolerate liver puncture, the “gold standard” examination. However, liver elastography can accurately reflect the degree of LS and is conducive to the quantitative comparison of LS differences among patients. Therefore, liver elastography was used in this study as an evaluation method for LS in patients with HFpEF.

According to the liver transient elastography assessment criteria defined by the guidelines (24), the LS threshold of normal people is 7.0 kPa (Supplementary Figure 5), which means when the LS value exceeds 7.0 kPa, it indicates significant liver fibrosis (≥F2). Roulot et al. (25) examined the “normal” liver hardness of 429 healthy subjects, whose average liver hardness was 5.5 ± 1.6 kPa. However, the results of our study showed that the average liver elasticity value of all HFpEF patients was 7.67 ± 1.03 kPa, significantly higher than the normal value, among which more than 2/3 had liver elasticity value >7.0 kPa, indicating a common prevalence of elevated LS in patients with HFpEF.

According to the results of this study, the higher the liver elasticity value of the group, the higher the NYHA class and the lgNT-proBNP, and there are significant differences among groups. It suggests that the degree of LS of HFpEF is related to the severity of heart failure when the primary liver disease is excluded, which is similar to the results of many previous researches. Hopper et al. (26) conducted liver elastography on 32 patients with chronic left heart failure and found that higher NYHA class was correlated with higher liver elasticity value. Colli et al. (27) performed liver elastography on 24 patients with acute decompensated heart failure before and after treatment, and found that with the improvement of clinical symptoms, liver elasticity value tended to decline, which was consistent with the improvement of cardiac function and NT-proBNP. Nishi et al. (28) demonstrated that liver elasticity value was significantly correlated with BNP and proposed that liver elasticity value could be used as an indicator reflecting the severity of heart failure. Taniguchi T et al. revealed that LS is a useful index for assessing systemic volume status and predicting the severity of HF, and that the presence of liver congestion at discharge is associated with worse outcomes in patients with HF (29). However, these studies lacked data on the population of HFpEF. Our study proved that increased LS was also strongly associated with HFpEF patients, indicating that not only systolic dysfunction, but also diastolic dysfunction may lead to increased LS. To further verify this correlation, we compared diastolic function and degree of left ventricular hypertrophy in different LS groups.

In this study, mitral annular e′ velocity, mean E/e′, left atrial volume index, and TR peak flow rate were used to evaluate left ventricular diastolic function in HFpEF, as recommended by the guideline. The results of our study showed that groups with different LS showed inter-group differences in these four indicators, especially in E/e′ and TR peak flow rate. Comparison of total diastolic function in the three groups showed that diastolic dysfunction became more common with the increase of LS, with the difference statistically significant. Left ventricular hypertrophy is another important pathophysiological change of HFpEF. The impaired active relaxation ability and decreased cardiac compliance are important causes of diastolic dysfunction (29). Our results showed that with the increase of LS, the left ventricular hypertrophy reflected by the LVMI increased. Our study demonstrated that the degree of LS of HFpEF was closely related to left ventricular diastolic function and left ventricular hypertrophy. The higher the LS, the worse the diastolic function as well as the left ventricular hypertrophy in patients with HFpEF. This is a new finding, and we suppose the reason for this association is that left ventricular diastolic dysfunction leads to pulmonary hypertension, which in turn leads to right ventricular hypertrophy and increased central venous pressure, leading to liver congestion. However, on the other hand, numerous studies have shown that endothelial cell dysfunction, as an important pathophysiological factor of HFpEF, is also greatly involved in the increase of LS (30–32), suggesting that increased LS and HFpEF may not be a single causal relationship, but the result of the joint action of some factors.

Therefore, we further investigated the correlation between LS of HFpEF and right cardiac function. We found that with the increase of liver elasticity value, the right ventricular systolic function represented by RVFAC and TAPSE became worse, while the right ventricular pressure and volume load represented by pulmonary systolic pressure, TR peak flow rate, and inferior vena cava diameter became heavier. The right heart has long been considered to play a secondary role in the maintenance of cardiac hemodynamics, and the effect of right cardiac function on HFpEF has been underestimated. Our study highlights the importance of right ventricular dysfunction in HFpEF, which is consistent with many recent studies. Chatterjee et al. (33) found that right heart dysfunction was very common in HFpEF by comparing right heart catheter measurement parameters, ultrasound parameters and prognostic indicators between HFpEF and normal subjects, and right cardiac dysfunction was the strongest predictor of prognosis in HFpEF. Pulmonary hypertension has been reported to be very common in HFpEF, accompanied by increased pulmonary artery-right ventricular resistance and decreased pulmonary artery compliance, confirming the presence of significant pulmonary vascular lesions in HFpEF (34, 35). On the other hand, the relationship between liver abnormalities and tricuspid regurgitation has been a research hotspot in recent years (36, 37). Our study demonstrated for the first time that the degree of LS in HFpEF was positively correlated with the severity of tricuspid regurgitation, and the correlation coefficient between liver elasticity value and TR peak flow rate was the largest, suggesting that tricuspid regurgitation was the most direct cause of increased LS.

However, it is worth noting that even in the no-RVD group, more than half (55%) of the patients had significant increased LS (liver elasticity > 7.0 kPa). This is an interesting finding, which indicates that increased LS in HFpEF is not entirely the result of RVD, on the contrary, as said before, is likely to be the joint action of inflammation, oxidative stress and vascular endothelial cell damage factors. This once again proved HFpEF is a systematic disease, and increased liver stiffness and myocardial stiffness may be the result of common factors acting on different organs.

Although woman is a risk factor for HFpEF, we found a significantly higher proportion of men in HFpEF combined with RVD than in HFpEF without RVD. Previous studies in HFrEF reported an association between males and right ventricular dysfunction (38, 39). RV volume is larger in men without heart failure, but EF is lower than women, and this difference may be related to sex hormone levels (40). Experimental studies have shown that male mice are more susceptible to the effects of stress overload than female mice, and this effect can be corrected by the reduction of testosterone levels (41). This also explains why the proportion of men in the first, second and third quartile groups in this study was gradually increased (although there was no significant difference), suggesting that gender may affect the degree of LS to a certain extent.

This study further explored the correlation between LS and short-term prognosis in patients with HFpEF. With the increase of LS, the prognosis of patients with HFpEF became worse, which was mainly manifested by an increase in the number of re-hospitalizations due to heart failure. MACE-free Kaplan Meier (K-M) survival curves showed that with the increase of follow-up days, the survival rate without MACE in the third quartile was significantly lower than that in the first and the second quartile. Multivariate Cox risk analysis showed that liver elasticity value, NYHA class, atrial fibrillation, and lgNT-proBNP were independently associated with poor prognosis of HFpEF. Our results prove that the degree of LS is closely related to the short-term prognosis of patients with HFpEF, and the more severe the degree of LS, the worse the short-term prognosis. More importantly, this study provides a theoretical support for this correlation, namely that the degree of LS reflects left ventricular diastolic function, left ventricular hypertrophy, and right cardiac function in patients with HFpEF. In other words, the degree of LS can predict the severity of HFpEF, and thus predict its clinical outcome. In terms of the predictive value of prognosis, liver elasticity value is better than NT-proBNP in some aspects. For example, NT-proBNP reflects the immediate severity of heart failure, vulnerable to cardiac functional state and change greatly. In contrast, as an indicator of liver stiffness, liver elasticity value is the result of long-term heart-liver influence, so it is more stable and can better reflect the long-term cardiac function status of patients. Therefore, liver elasticity value as a prognostic index has a certain promotion value.

There are some limitations in this study. First, assessments of diastolic function, ventricular hypertrophy, and right cardiac function were based on echocardiographic indicators, lacking hemodynamic indicators such as cardiac MRI and invasive catheter measurements. Secondly, the poor prognosis in this study mainly refers to the number of re-hospitalizations due to heart failure rather than death. Due to the limitation of follow-up time and sample size, we could not clearly establish the correlation between LS and mortality.

## Conclusion

Increased LS is common in patients with HFpEF. The degree of LS reflected by liver elastography is not associated with LVEF, but closely correlated with left ventricular diastolic function, left ventricular hypertrophy, and right cardiac function in patients with HFpEF. Male, atrial fibrillation, higher NYHA class, and higher liver elasticity value are independently associated with RVD in HFpEF. Moreover, Increased LS is associated with worse short-term (<1 year) prognosis for HFpEF. Future studies with larger sample sizes, longer follow-up, and the introduction of cardiac MR and invasive cardiac catheterization are warranted to further explore the association between LS and HFpEF.

## Data Availability Statement

The original contributions presented in the study are included in the article/Supplementary Material, further inquiries can be directed to the corresponding author.

## Ethics Statement

The studies involving human participants were reviewed and approved by Ethics Committee of Suzhou Dushu Lake Hospital. The patients/participants provided their written informed consent to participate in this study.

## Author Contributions

JZ designed the study and collected case data. MX analyzed the data and wrote the article. TC and YZ gave guidance to the development of this study and the writing of the article. All authors contributed to the article and approved the submitted version.

## Funding

This work was supported by grants from National Natural Science Foundation of China (Nos. 81873486 and 81770327), Natural Scientific Fund of Jiangsu province (No. BK20161226), Jiangsu Province's Key Provincial Talents Program (No. ZDRCA2016043), Jiangsu Province's 333 High-Level Talents Project (No. BRA2017539), and Jiangsu Provincial Medical Innovation Team (No. CXTDA2017009). The funders had no roles in study design, data collection and analysis, decision to publish, or preparation of the manuscript.

## Conflict of Interest

The authors declare that the research was conducted in the absence of any commercial or financial relationships that could be construed as a potential conflict of interest.

## Publisher's Note

All claims expressed in this article are solely those of the authors and do not necessarily represent those of their affiliated organizations, or those of the publisher, the editors and the reviewers. Any product that may be evaluated in this article, or claim that may be made by its manufacturer, is not guaranteed or endorsed by the publisher.

## Supplementary Material

Supplementary Figure 1 Scatter distribution of liver elastography values (LEV) and various laboratory indicators. **(A)** LEV and A/G; **(B)** LEV and Tbil; **(C)** LEV and Dbil; **(D)** LEV and Ibil; **(E)** LEV and ALP; **(F)** LEV and GGT; **(G)** LEV and lgNT-proBNP.

Supplementary Figure 2 Scatter distribution of liver elastography values (LEV) and various left ventricular parameters. **(A)** LEV and ventricular septal e′; **(B)** LEV and lateral wall e′; **(C)** LEV and average E/e′; **(D)** LEV and LAVI; **(E)** LEV and TR peak flow rate; **(F)** LEV and LVMI (Male); **(G)** LEV and LVMI (Female).

Supplementary Figure 3 Scatter distribution of liver elastography values (LEV) and various right heart parameters. **(A)** LEV and right atrial diameter; **(B)** LEV and RVFAC; **(C)** LEV and TAPSE; **(D)** LEV and pulmonary systolic pressure; **(E)** LEV and TR peak flow rate.

Supplementary Figure 4 Detailed results of Kaplan-Meier analyses using Log-rank Test.

Supplementary Figure 5 Grading of liver fibrosis based on liver elastography value according to guidelines.

Supplementary Table 1 Comparison of LEV between HFrEF and HFpEF.

Supplementary Table 2 Comparison of clinical features among liver elastography value (LEV) quartiles.

Supplementary Table 3 Univariate and multivariate cox proportional hazard analysis of predicting MACEs in patients with HFpEF.
