# Supplementary figures and images for: Correlation Between Liver Stiffness and Diastolic Function, Left Ventricular Hypertrophy, and Right Cardiac Function in Patients With Ejection Fraction Preserved Heart Failure
Source: Front Cardiovasc Med. 2021 Nov 25;8:748173. doi: 10.3389/fcvm.2021.748173 (PMC8655684; doi:10.3389/fcvm.2021.748173)

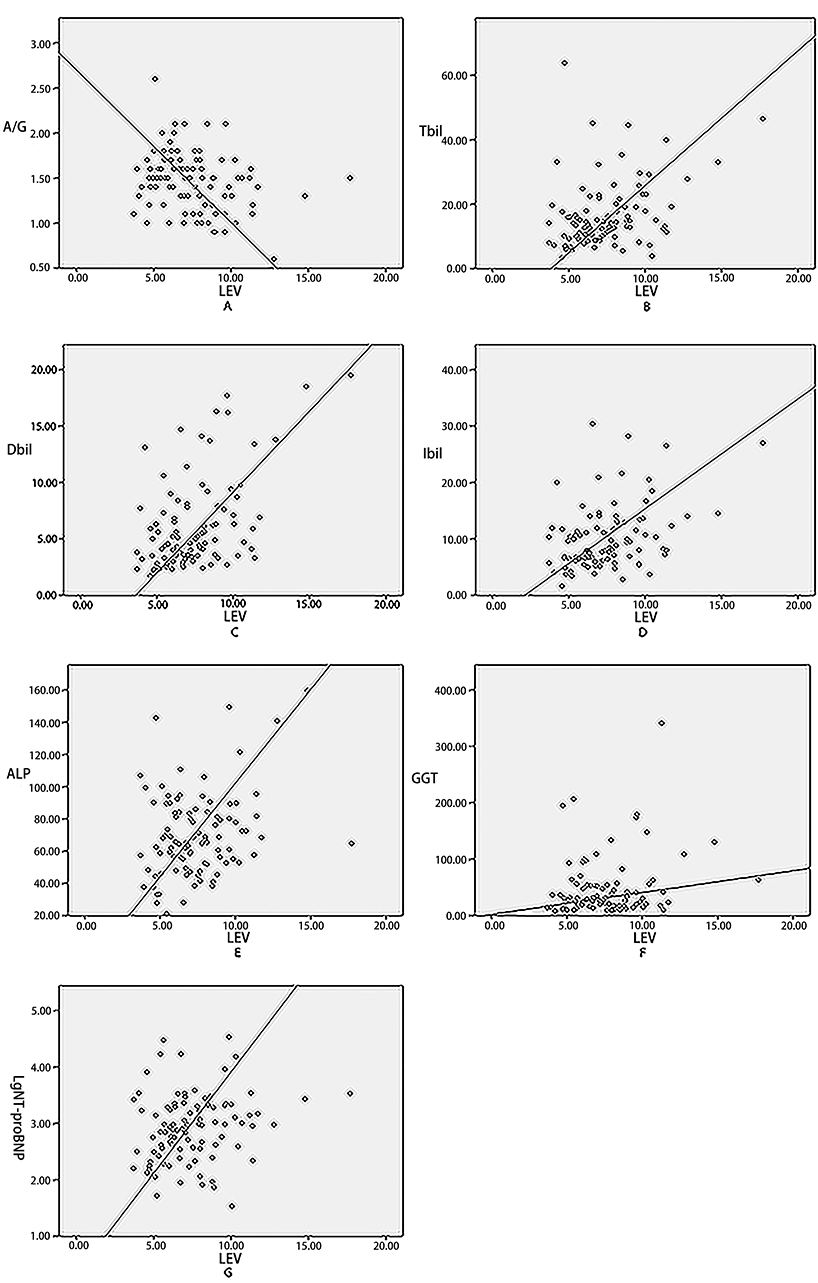

Supplement: Supplementary Figure 1 — Scatter distribution of liver elastography values (LEV) and various laboratory indicators. (A) LEV and A/G; (B) LEV and Tbil; (C) LEV and Dbil; (D) LEV and Ibil; (E) LEV and ALP; (F) LEV and GGT; (G) LEV and lgNT-proBNP. [file Image_1.PNG]

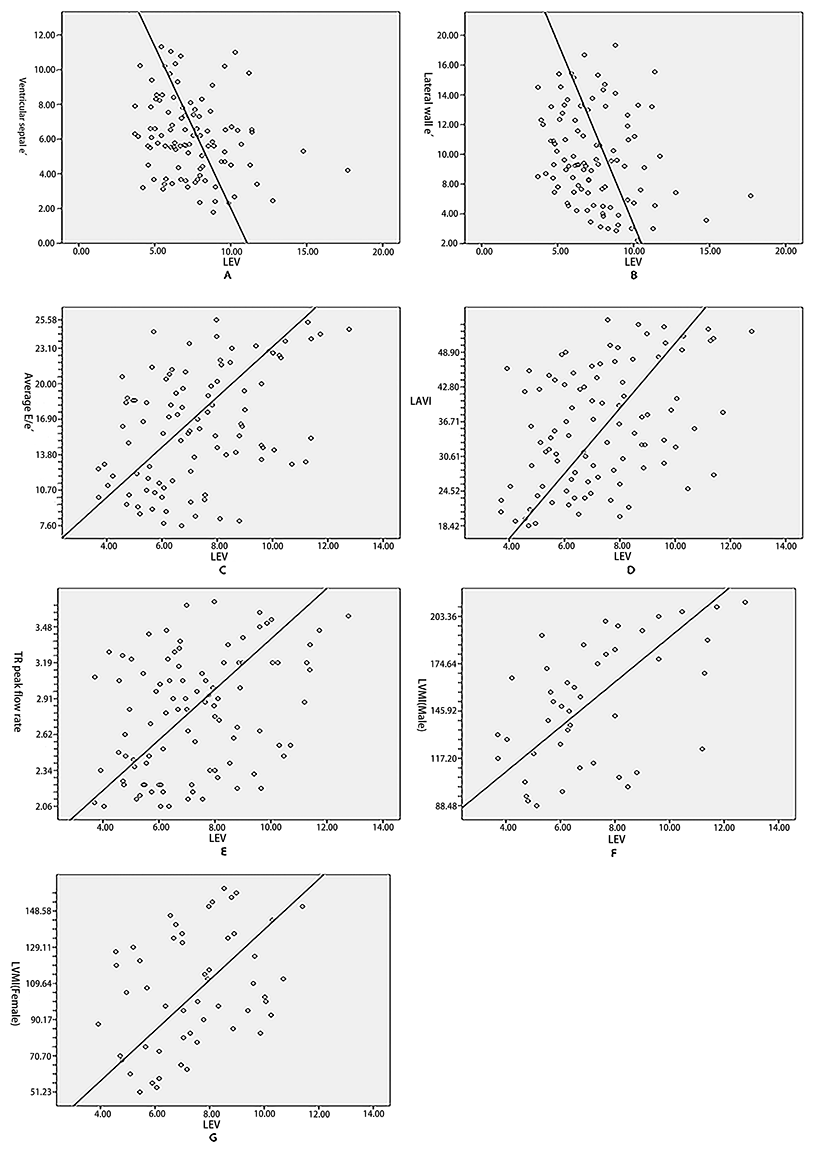

Supplement: Supplementary Figure 2 — Scatter distribution of liver elastography values (LEV) and various left ventricular parameters. (A) LEV and ventricular septal e′; (B) LEV and lateral wall e′; (C) LEV and average E/e′; (D) LEV and LAVI; (E) LEV and TR peak flow rate; (F) LEV and LVMI (Male); (G) LEV and LVMI (Female). [file Image_2.PNG]

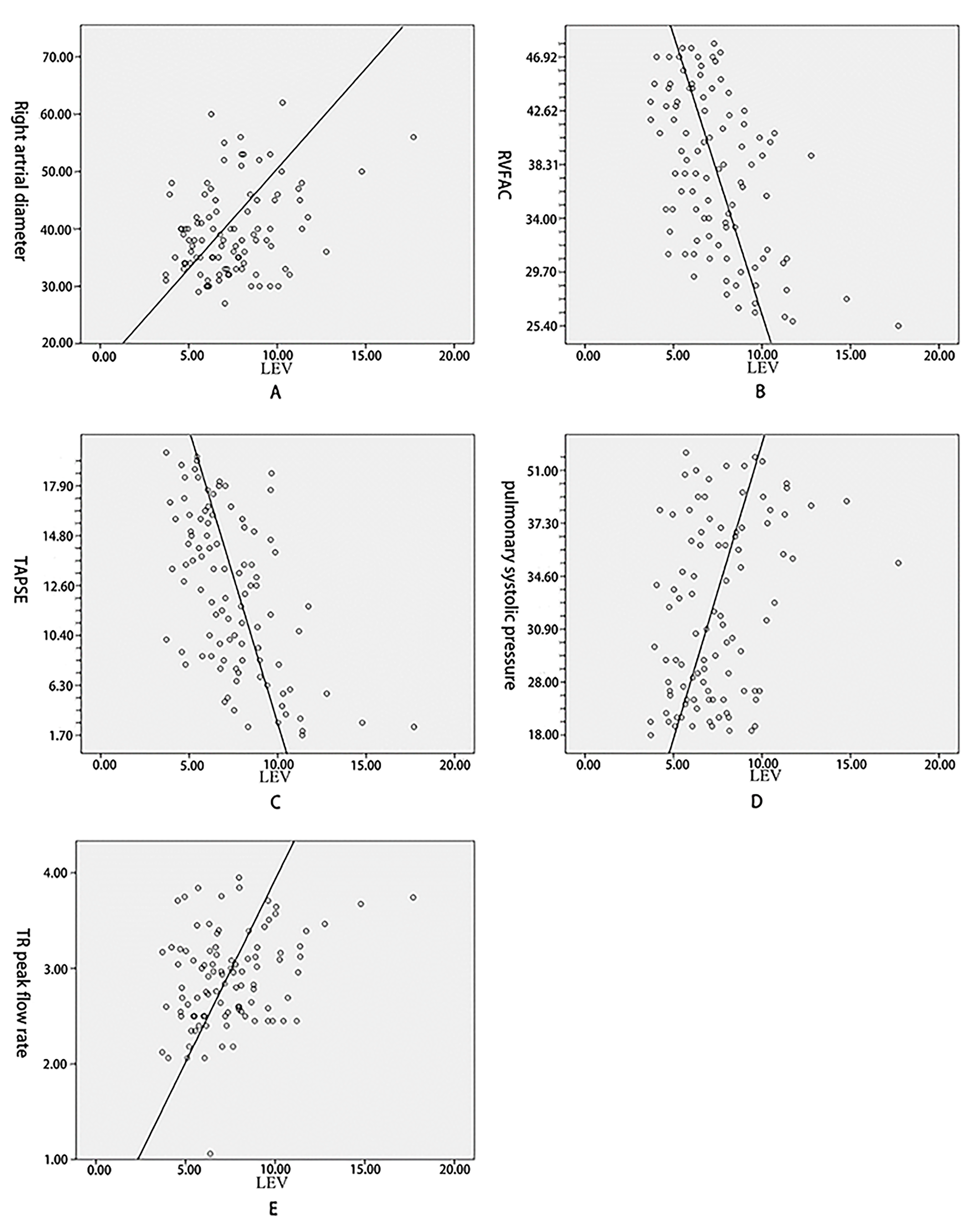

Supplement: Supplementary Figure 3 — Scatter distribution of liver elastography values (LEV) and various right heart parameters. (A) LEV and right atrial diameter; (B) LEV and RVFAC; (C) LEV and TAPSE; (D) LEV and pulmonary systolic pressure; (E) LEV and TR peak flow rate. [file Image_3.PNG]

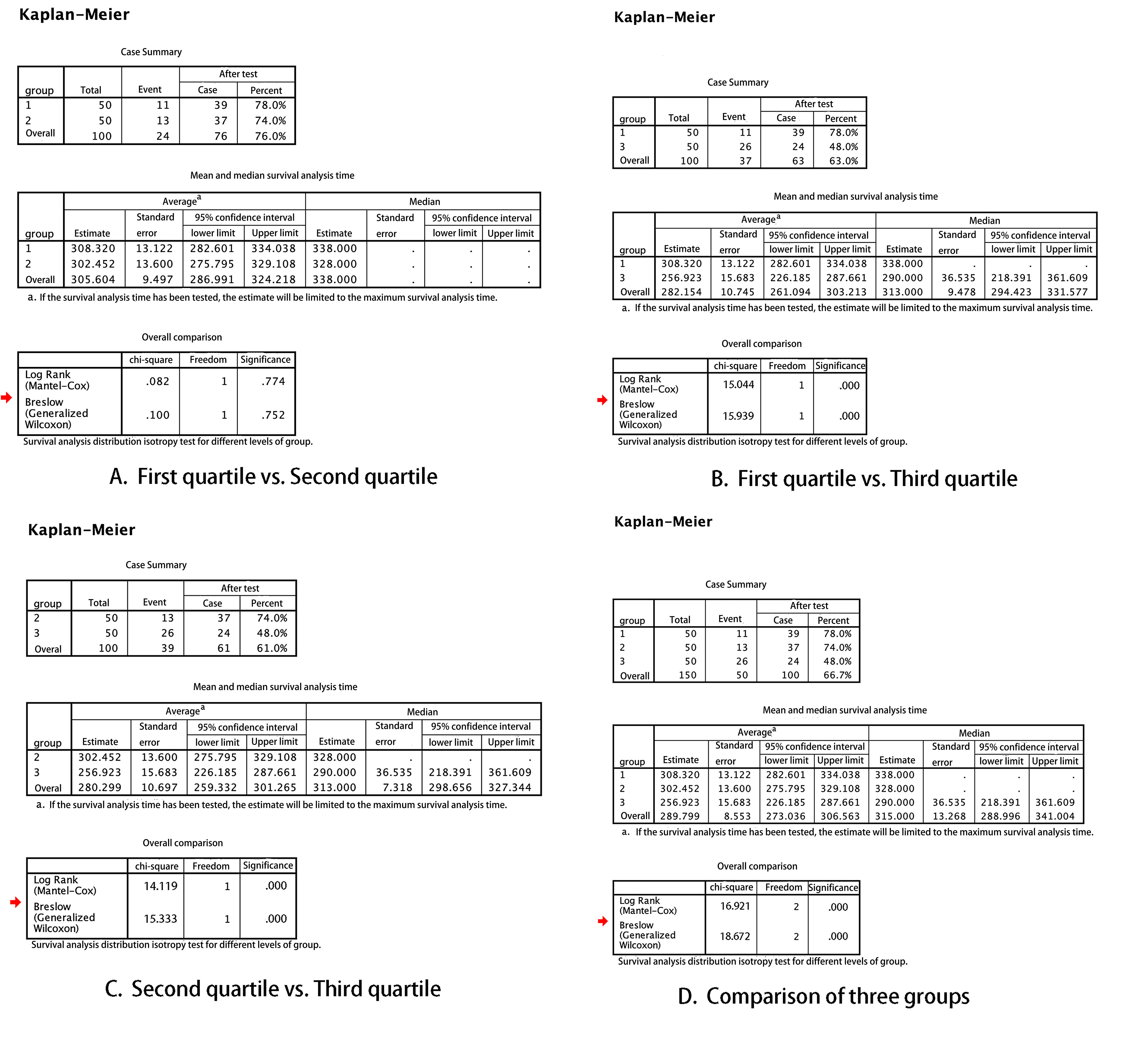

Supplement: Supplementary Figure 4 — Detailed results of Kaplan-Meier analyses using Log-rank Test. [file Image_4.PNG]

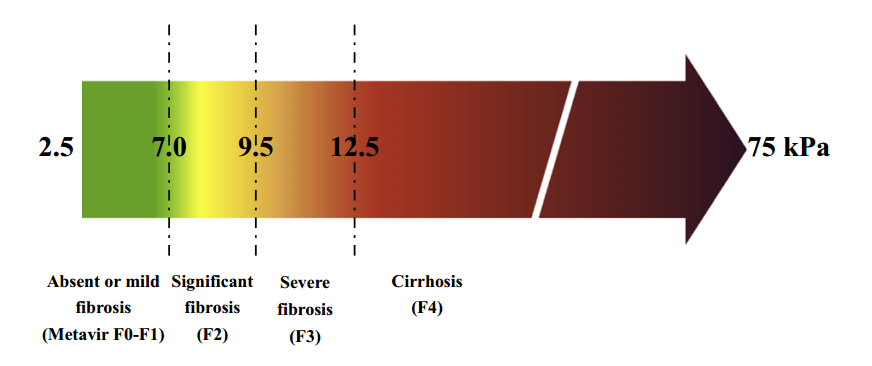

Supplement: Supplementary Figure 5 — Grading of liver fibrosis based on liver elastography value according to guidelines. [file Image_5.PNG]
