# Supplementary material for: Correlation Between Liver Stiffness and Diastolic Function, Left Ventricular Hypertrophy, and Right Cardiac Function in Patients With Ejection Fraction Preserved Heart Failure
Source: Front Cardiovasc Med. 2021 Nov 25;8:748173. doi: 10.3389/fcvm.2021.748173 (PMC8655684; doi:10.3389/fcvm.2021.748173)
Supplement: Supplementary Table 1 — Comparison of LEV between HFrEF and HFpEF. [file Table_1.DOCX]

| **Supplementary Table 1: Comparison of LEV between HFrEF and HFpEF** | | | |
| --- | --- | --- | --- |
|  | **HFrEF** | **HFpEF** | **P value** |
| **N** | 30 | 30 | - |
| **Male/Female** | 15/15 | 15/15 | - |
| **Age, yrs** | 62.7±8.4 | 66.27±9.5 | 0.243 |
| **LEV, kPa** | 7.85±0.19 | 8.27±0.24 | 0.154 |

*LEV*, liver elastography value.
