# Supplementary material for: Correlation Between Liver Stiffness and Diastolic Function, Left Ventricular Hypertrophy, and Right Cardiac Function in Patients With Ejection Fraction Preserved Heart Failure
Source: Front Cardiovasc Med. 2021 Nov 25;8:748173. doi: 10.3389/fcvm.2021.748173 (PMC8655684; doi:10.3389/fcvm.2021.748173)
Supplement: Supplementary Table 2 — Comparison of clinical features among liver elastography value (LEV) quartiles. [file Table_2.docx]

| **Supplementary Table 2** Comparison of clinical features among liver elastography value (LEV) quartiles | | | | | |
| --- | --- | --- | --- | --- | --- |
|  | All patients  （N=150） | First quartile  LEV<7.15  (N=50) | Second quartile  7.15≤LEV<8.30  （N=50） | Third quartile  8.30≤LEV  （N=50） | P value |
| LEV (kPa) | 7.67±1.03 | 6.63±0.71 **#** | 7.51±0.69 ***** | 10.10±0.86 ***#** | **<0.001** |
| Age（years） | 68.04±13.2 | 66.3±12.2 | 68.6±14.7 | 69.2±10.0 | 0.346 |
| Male gender (n,%) | 65（43.3） | 18（36.0） | 20（40.0） | 27（54.0） | 0.102 |
| BMI | 24.85±4.35 | 24.22±3.93 | 24.45±4.17 | 24.56±4.69 | 0.436 |
| LVEF (%) | 60.7±13.0 | 59.5±12.2 | 60.4±14.0 | 61.7±14.2 | 0.635 |
| LVDd | 53.4±9.6 | 51.6±9.3 | 53.5±10.2 | 55.2±9.9 | 0.147 |
| NYHA grade II/III/IV (n) | 25/44/81 | 15/16/19 | 8/15/27 | 2/13/35 ***** | **0.022** |
| eGFR (ml/min) | 78.84±41.35 | 82.57±43.95 | 75.91±35.72 | 79.81±37.27 | 0.773 |
| **Co-morbidity** |  |  |  |  |  |
| Hypertension (n,%) | 106（70.7） | 28（56.0） | 37（74.0） | 41（82.0） | 0.057 |
| Diabetes (n,%) | 50（33.3） | 11（22.0）**#** | 19（38.0）***** | 20（40.0）***** | **0.014** |
| Dyslipidaemia (n,%) | 33（22.0） | 9（18.0） | 13（26.0） | 11（22.0） | 0.430 |
| CAD (n,%) | 40（26.7） | 13（26.0） | 12（24.0） | 15（30.0） | 0.372 |
| Atrial fibrillation (n,%) | 50（33.3） | 10（20.0）**#** | 18（36.0）***** | 22（44.0）***** | **0.010** |
| CKD (n,%) | 21（14.0） | 8（16.0） | 7（14.0） | 6（12.0） | 0.908 |
| COPD (n,%) | 11（7.3） | 5（10.0） | 3（6.0） | 3（6.0） | 0.837 |
| Anaemia (n,%) | 14（9.3） | 3（6.0） | 5（10.0） | 6（12.0） | 0.610 |
| Smoking (n,%) | 37（24.7） | 11（22.0） | 14（28.0） | 12（24.0） | 0.817 |
| Drinking (n,%) | 18（12.0） | 5（10.0） | 7（14.0） | 6（12.0） | 0.904 |
| **Laboratory data** |  |  |  |  |  |
| lg NT-proBNP | 2.81±0.68 | 2.63±0.65 **#** | 2.84±0.44 ***** | 3.05±0.71 ***#** | **0.027** |
| Hb（g/L） | 125±20 | 125±17 | 126±19 | 124±19 | 0.901 |
| PLT（10^9/L） | 181±69 | 183±70 | 184±65 | 176±50 | 0.862 |
| Na（mmol/L） | 141.2±3.1 | 141.1±2.2 | 141.4±2.7 | 141.4±3.5 | 0.924 |
| K（mmol/L） | 3.86±0.39 | 3.85±0.38 | 3.93±0.42 | 3.81±0.41 | 0.234 |
| Ca（mmol/L） | 2.19±0.12 | 2.21±0.13 | 2.22±0.11 | 2.18±0.12 | 0.356 |
| UA（umol/L） | 368±137 | 363±115 | 357±158 | 388±142 | 0.646 |
| Tlb (g/L) | 69.1±9.3 | 67.1±6.9 | 66.1±6.8 | 71.1±13.7 | 0.097 |
| Alb（g/L） | 39.5±5.2 | 40.2±4.9 | 39.4±4.6 | 38.2±5.7 | 0.279 |
| Glb（g/L） | 28.4±5.1 | 26.9±4.4 | 26.7±4.5 | 28.8±5.9 | 0.200 |
| A/G | 1.47±0.32 | 1.53±0.31 | 1.52±0.30 | 1.33±0.33 ***#** | **0.016** |
| TBil (umol/L) | 16.4±9.4 | 14.0±10.9 | 14.8±8.2 | 20.0±10.8 ***#** | **0.033** |
| DBil (umol/L) | 5.9±3.6 | 4.6±2.7 | 5.4±3.1 | 8.2±5.0 ***#** | **0.002** |
| IBil (umol/L) | 10.4±5.5 | 8.0±3.8 | 9.3±5.4 | 12.6±6.4 ***#** | **0.002** |
| ALT (U/L) | 25.7±13.6 | 32.9±48.7 | 23.4±18.6 | 18.7±8.9 | 0.164 |
| AST (U/L) | 25.1±12.4 | 31.5±41.5 | 23.2±11.6 | 22.6±9.7 | 0.291 |
| ALP (U/L) | 71.2±24.5 | 67.1±27.1 | 68.0±19.9 | 76.9±29.2 | 0.173 |
| GGT (U/L) | 45.6±51.4 | 43.8±61.1 | 47.4±28.4 | 57.6±69.5 | 0.325 |
| α-HBDH (U/L) | 158.8±42.2 | 156.0±51.3 | 159.3±40.6 | 161.8±38.3 | 0.865 |
| LDH (U/L) | 196.3±54.7 | 195.8±64.8 | 193.2±45.0 | 199.8±43.2 | 0.874 |
| CK (U/L) | 82.4±49.6 | 85.6±61.7 | 81.4±48.5 | 80.7±39.6 | 0.917 |
| TG (mmol/L) | 1.37±0.59 | 1.54±0.77 | 1.27±0.77 | 1.17±0.59 | 0.113 |
| TC (mmol/L) | 4.14±1.22 | 4.31±1.20 | 4.03±1.17 | 4.05±1.28 | 0.581 |
| HDL-C (mmol/L) | 1.02±0.33 | 1.00±0.27 | 1.04±0.40 | 1.04±0.34 | 0.867 |
| LDL-C (mmol/L) | 2.45±1.12 | 2.60±1.09 | 2.33±0.97 | 2.40±1.14 | 0.577 |
| Lp(a) (mmol/L) | 193.4±138.9 | 208.7±194.5 | 197.7±186.3 | 170.7±126.5 | 0.655 |
| ApoA_1_ (mmol/L) | 1.16±0.22 | 1.17±0.20 | 1.17±0.20 | 1.15±0.23 | 0.904 |
| ApoB (mmol/L) | 0.81±0.27 | 0.85±0.29 | 0.77±0.24 | 0.77±0.29 | 0.404 |
| ApoA_1_/ApoB | 1.66±0.63 | 1.49±0.42 | 1.65±0.57 | 1.70±0.73 | 0.339 |
| FT_3_ (pmol/ml) | 4.39±1.12 | 4.36±0.96 | 4.31±0.83 | 4.46±1.70 | 0.885 |
| FT_4_ (pmol/ml) | 17.3±4.5 | 17.5±2.6 | 16.2±3.2 | 18.0±5.6 | 0.176 |
| TSH (mIU/L) | 3.23±2.35 | 2.69±1.53 | 3.66±4.54 | 3.03±2.45 | 0.445 |
| BMI, body mass index; LVEF, left ventricular ejection fraction; eGFR, estimated glomerular filtration rate; CAD,  coronary artery disease; CKD, chronic kidney disease; COPD, chronic obstructive pulmonary disease; Hb, hemoglobin;  PLT, platelet; UA, Uric acid; Tlb, total protein; Alb, albumin; Glb, globulin; TBil, total bilirubin; DBil, direct bilirubin;  IBil, indirect bilirubin; ALT, alanine aminotransferase; AST, aspartate aminotransferase; ALP, alkaline phosphatase; GGT,  Glutamyltranspeptidase; α-HBDH, α-hydroxybutyrate dehydrogenase; LDH, lactate dehydrogenase; CK, creatine kinase;  TG, total triglyceride; TC, total cholesterol; HDL, high density cholesterol; LDL, low density cholesterol; TSH, Thyrotropic  Hormone; LVDd, left ventricular end-diastolic diameter. | | | | | |
| *****：P<0.05 vs. the first quartile  **#**：P<0.05 vs. the second quartile | | | | | |
