# Supplementary material for: Correlation Between Liver Stiffness and Diastolic Function, Left Ventricular Hypertrophy, and Right Cardiac Function in Patients With Ejection Fraction Preserved Heart Failure
Source: Front Cardiovasc Med. 2021 Nov 25;8:748173. doi: 10.3389/fcvm.2021.748173 (PMC8655684; doi:10.3389/fcvm.2021.748173)
Supplement: Supplementary Table 3 — Univariate and multivariate cox proportional hazard analysis of predicting MACEs in patients with HFpEF. [file Table_3.DOCX]

| **Supplementary Table 3** Univariate and multivariate cox proportional hazard analysis of predicting MACEs in patients with HFpEF. | | | | | | | |
| --- | --- | --- | --- | --- | --- | --- | --- |
|  | Univariate analysis | | |  | Multivariate analysis | | |
|  | HR | 95% CI | P value |  | HR | 95% CI | P value |
| Age (years) | 1.045 | 1.021-1.061 | **<0.0001** |  | 1.012 | 0.986-1.032 | 0.114 |
| Male | 1.065 | 0.785-1.427 | 0.5482 |  |  |  |  |
| BMI, kg/m^2^ | 0.942 | 0.902-0.980 | **0.0015** |  | 0.972 | 0.913-1.034 | 0.205 |
| NYHAclass（III-IV vs. II） | 3.985 | 2.243-8.334 | **<0.0001** |  | 2.792 | 1.443-5.704 | **0.002** |
| Hypertension | 0.874 | 0.670-1.183 | 0.2350 |  |  |  |  |
| Diabetes | 1.568 | 1.085-2.032 | **0.0007** |  | 1.216 | 0.993-1.572 | 0.096 |
| Hyperlipidemia | 1.034 | 0.736-1.417 | 0.6995 |  |  |  |  |
| Atrial fibrillation | 1.927 | 1.313-2.541 | **<0.0001** |  | 1.135 | 1.032-1.304 | **0.029** |
| Smoke | 1.322 | 1.141-1.536 | **0.0010** |  | 1.101 | 0.986-1.215 | 0.116 |
| eGFR, ml/min | 0.970 | 0.952-0.985 | **0.0024** |  | 0.990 | 0.963-1.011 | 0.089 |
| lg NT-proBNP | 1.485 | 1.322-1.704 | **<0.0001** |  | 1.189 | 1.010-1.356 | **0.015** |
| Hb, g/L | 0.897 | 0.842-0.968 | **0.0045** |  | 0.939 | 0.872-1.005 | 0.101 |
| PLT, 10^^^9/L | 0.995 | 0.989-1.001 | 0.1104 |  |  |  |  |
| Tlb, g/L | 0.988 | 0.963-1.018 | 0.2245 |  |  |  |  |
| Alb, g/L | 0.872 | 0.828-0.954 | **0.0023** |  | 0.945 | 0.887-1.018 | 0.102 |
| Glb, g/L | 1.012 | 0.991-1.023 | 0.2540 |  |  |  |  |
| A/G | 0.972 | 0.944-1.014 | 0.0724 |  |  |  |  |
| Tbil, umol/L | 0.954 | 0.713-1.141 | 0.5113 |  |  |  |  |
| Dbil, umol/L | 0.932 | 0.876-1.102 | 0.4884 |  |  |  |  |
| Ibil, mg/dl | 0.963 | 0.915-1.224 | 0.6010 |  |  |  |  |
| ALT, U/L | 1.003 | 0.992-1.103 | 0.7529 |  |  |  |  |
| AST, U/L | 1.001 | 0.989-1.110 | 0.6932 |  |  |  |  |
| ALP, U/L | 1.024 | 0.994-1.118 | 0.7004 |  |  |  |  |
| GGT, U/L | 0.992 | 0.982-1.012 | 0.6472 |  |  |  |  |
| α-HBDH, U/L | 0.982 | 0.955-1.168 | 0.8221 |  |  |  |  |
| LDH, U/L | 1.000 | 0.991-1.009 | 0.4128 |  |  |  |  |
| TG，mmol/L | 1.015 | 0.992-1.214 | 0.5344 |  |  |  |  |
| TC, mmol/L | 1.032 | 0.998-1.054 | 0.0631 |  |  |  |  |
| HDL-C, mmol/L | 0.972 | 0.933-1.032 | 0.1002 |  |  |  |  |
| LDL-C, mmol/L | 1.096 | 1.011-1.162 | **0.0013** |  | 1.035 | 0.994-1.070 | 0.112 |
| Na, mmol/L | 0.998 | 0.987-1.013 | 0.9175 |  |  |  |  |
| K, mmol/L | 0.994 | 0.982-1.020 | 0.8771 |  |  |  |  |
| Ca, mmol/L | 1.087 | 0.993-1.192 | 0.0956 |  |  |  |  |
| FT_3_, pmol/ml | 1.023 | 0.989-1.298 | 0.4896 |  |  |  |  |
| FT_4_, pmol/ml | 1.029 | 0.982-1.311 | 0.5122 |  |  |  |  |
| TSH, mIU/L | 0.990 | 0.975-1.009 | 0.6120 |  |  |  |  |
| LVEF, % | 0.988 | 0.976-1.004 | 0.1014 |  |  |  |  |
| LEV, Kpa | 1.305 | 1.152-1.485 | **<0.0001** |  | 1.208 | 1.115-1.352 | **0.002** |
